# Characterizing the Adoption and Experiences of Users of Artificial Intelligence–Generated Health Information in the United States: Cross-Sectional Questionnaire Study

**DOI:** 10.2196/55138

**Published:** 2024-08-14

**Authors:** Oluwatobiloba Ayo-Ajibola, Ryan J Davis, Matthew E Lin, Jeffrey Riddell, Richard L Kravitz

**Affiliations:** 1 Keck School of Medicine of the University of Southern California Los Angeles, CA United States; 2 Department of Head and Neck Surgery David Geffen School of Medicine at University of California Los Angeles Los Angeles, CA United States; 3 Department of Emergency Medicine Keck School of Medicine of the University of Southern California Los Angeles, CA United States; 4 Division of General Medicine University of California Davis Sacramento, CA United States

**Keywords:** artificial intelligence, ChatGPT, health information, patient information-seeking, online health information, health literacy, ResearchMatch, users, diagnosis, decision-making, cross-sectional, survey, surveys, adoption, utilization, AI, less-educated, poor health, worse health, experience, experiences, user, users, non user, non users, AI-generated, health information, implication, implications, medical practice, medical practices, public health, descriptive statistics, t test, t tests, chi-square test, chi-square tests, health-seeking behavior, health-seeking behaviors, patient-provider, interaction, interactions, patient, patients

## Abstract

**Background:**

OpenAI’s ChatGPT is a source of advanced online health information (OHI) that may be integrated into individuals’ health information-seeking routines. However, concerns have been raised about its factual accuracy and impact on health outcomes. To forecast implications for medical practice and public health, more information is needed on who uses the tool, how often, and for what.

**Objective:**

This study aims to characterize the reasons for and types of ChatGPT OHI use and describe the users most likely to engage with the platform.

**Methods:**

In this cross-sectional survey, patients received invitations to participate via the ResearchMatch platform, a nonprofit affiliate of the National Institutes of Health. A web-based survey measured demographic characteristics, use of ChatGPT and other sources of OHI, experience characterization, and resultant health behaviors. Descriptive statistics were used to summarize the data. Both 2-tailed *t* tests and Pearson chi-square tests were used to compare users of ChatGPT OHI to nonusers.

**Results:**

Of 2406 respondents, 21.5% (n=517) respondents reported using ChatGPT for OHI. ChatGPT users were younger than nonusers (32.8 vs 39.1 years, *P*<.001) with lower advanced degree attainment (BA or higher; 49.9% vs 67%, *P*<.001) and greater use of transient health care (ED and urgent care; *P*<.001). ChatGPT users were more avid consumers of general non-ChatGPT OHI (percentage of weekly or greater OHI seeking frequency in past 6 months, 28.2% vs 22.8%, *P*<.001). Around 39.3% (n=206) respondents endorsed using the platform for OHI 2-3 times weekly or more, and most sought the tool to determine if a consultation was required (47.4%, n=245) or to explore alternative treatment (46.2%, n=239). Use characterization was favorable as many believed ChatGPT to be just as or more useful than other OHIs (87.7%, n=429) and their doctor (81%, n=407). About one-third of respondents requested a referral (35.6%, n=184) or changed medications (31%, n=160) based on the information received from ChatGPT. As many users reported skepticism regarding the ChatGPT output (67.9%, n=336), most turned to their physicians (67.5%, n=349).

**Conclusions:**

This study underscores the significant role of AI-generated OHI in shaping health-seeking behaviors and the potential evolution of patient-provider interactions. Given the proclivity of these users to enact health behavior changes based on AI-generated content, there is an opportunity for physicians to guide ChatGPT OHI users on an informed and examined use of the technology.

## Introduction

### Background

The internet is a highly trafficked source of health information, with over half of US adults polled in 2019 reporting the use of search engines and social media for health-related purposes [1,2]. With increasing ease of access to online health information (OHI), patients no longer rely exclusively on physicians for medical information, as many seek web-based guidance for understanding and managing personal health concerns [3-5].

On December 22, 2022, OpenAI released ChatGPT, their GPT-4 technology [6]. ChatGPT is a large language model (LLM) artificial intelligence (AI) trained on vast text data to generate human-like responses to text queries. As ChatGPT positions itself as a formidable alternative to conventional internet search engines, its capability to generate expert “human” conversations and responses continues to diversify and strengthen as the technology is improved through mass use [7,8]. Within 2 months it amassed 100 million unique users, marking the fastest online platform adoption in history [9].

ChatGPT has demonstrated proficiency in performing tasks on par with, and sometimes surpassing, physicians [10,11]. Ayers et al [12] revealed that ChatGPT could answer patient health questions on social media platforms more empathetically and effectively than some doctors. Another study highlighted the LLM’s competency in providing predominantly accurate information for health queries spanning over 17 specialties [13].

Nevertheless, ChatGPT’s primary objective—to produce human-like text—does not guarantee the accuracy of medical information. Considerable prior research has emphasized the assessment of the quality of ChatGPT’s responses to simulated health inquiries, further suggesting reliance on incorrect health advice may cause harm due to mismanagement or delays [12,14-19]. Thus, many health care professionals have encouraged caution when considering adopting the technology for patient advice and incorporation into practice [20,21].

As ChatGPT’s popularity soars, patients will likely integrate this tool into their health information-seeking routine. Notably, younger patients with more severe health conditions and limited health care system interactions have shown a propensity for OHI use and may tend to be early adopters of ChatGPT for health purposes [22]. Identifying the characteristics of early ChatGPT adopters may provide insight into who may benefit most from tailored guidance on appropriate use and potential risks of ChatGPT OHI. Further, understanding the purposes of patient use and the resultant health behaviors may help physicians as they support patients in their pursuit of accurate and reliable information to support their health care decisions.

Previous research has explored OHI-seeking behavior on other popular media such as YouTube and Facebook, however, minimal focus has been placed on the users of ChatGPT OHI [23-26]. Of the few studies that have examined the nature and experiences of patients actually using this OHI platform, none have explored the characteristics that differentiate ChatGPT OHI seekers from general OHI users [27]. This study aims to not only delineate the demographics and use characteristics of ChatGPT OHI-seekers, but also characterize user experience and subsequent action based on the information received.

### Objectives

This study aimed to address these knowledge gaps by posing the following 4 research questions:

- RQ1: How do ChatGPT OHI adopters’ demographic characteristics compare to nonusers?
- RQ2: How do ChatGPT OHI users characterize the purpose and frequency of their use?
- RQ3: How do users characterize the ease, understanding, and usefulness of ChatGPT OHI?
- RQ4: How do ChatGPT users use information derived from the tool?

## Methods

### Ethical Considerations

The University of Southern California Institutional Review Board approved this cross-sectional survey on human participants (UP# 23-00390).

This featured an information sheet that explained that the study aimed to record their use of OHI, their participation was voluntary, the survey would take up to 10 minutes, and their data would be completely anonymous (no identifiers were used). This also outlined privacy and confidentiality protections, including the use of digital and physical barriers to data vulnerability.

### Model Adaptation and Questionnaire Creation

Given the nascent nature of research on patient’s experiences with ChatGPT, we were unable to use an existing questionnaire. As such, we created a novel survey instrument by adapting several sources with previously collected valid evidence. The final questionnaire is available as Multimedia Appendix 1. Our approach was informed in part by the Health Beliefs Model, which identifies factors associated with the adoption of health-related behaviors (Multimedia Appendix 2) [28]. The model posits that adoption of a given health-related behavior is affected by perceived susceptibility to illness, severity of the issue, confidence in one’s ability to perform the behavior (self-efficacy), and perceived benefits and barriers to completing the desired health action. “Cues to Action” from events or other people may also spur the behavior. We surmised that younger patients, patients in worse health, those with more OHI experience, and those with acute health concerns (cues) would be most likely to report ChatGPT use in the first place and to use the tool more frequently. Multimedia Appendix 1 depicts the Health Beliefs Model adapted for predicting the use of ChatGPT.

Using a REDCap (Research Electronic Data Capture) questionnaire, we collected demographic details, including age, race, and preferred language [29]. We assessed sociodemographic factors via educational level, household income, and location of primary health care access [30]. Unfortunately, gender was inadvertently omitted in the initial survey. We rectified this oversight via an abbreviated separate second survey wave, wherein gender information was successfully captured. The purpose of this sample was to estimate the gender distribution of the population. The small sample size (n=137) constrained meaningful group comparisons.

Health literacy was assessed using the eHealth Literacy Scale, scored with a 5-point scale from 1=strongly disagree to 5=strongly agree [31]. A total of 4 of the 8 items from the original scale were retained, corresponding to the respondent’s ability to find and use health resources on the web, distinguish source quality, and make decisions based on the information they receive. Self-reported health status was assessed with the 5-point question: “How would you rate your health? (excellent, very good, good, fair, or poor)” [32].

The frequency of general OHI use was assessed using a list of popular sources of OHI, adapted from Zhang et al [33]. These included internet search engines, online encyclopedia sites, online health sites (eg, WebMD, MedlinePlus, and MayoClinic), online forums (eg, Reddit subgroups, Facebook groups, and specialized health organization forums), and question-and-answer sites. Survey progression depended on respondents identifying ChatGPT as one of their OHI channels. An attention question was included to screen out respondents who were not attending to the task at hand.

We evaluated perceived severity by inquiring how severe a problem usually is before the need for OHI arises. Perceived benefits were assessed with questions asking patients to indicate whether they agreed with multiple statements on a scale of strongly disagree (1) to strongly agree (5). These statements were as follows: “It is easy to use ChatGPT for the purpose of getting online health information,” “The information received from ChatGPT is easy to understand,” and “The online health information I receive from ChatGPT is relevant to my specific needs.”

Of the above, the last statement was adapted from Murray et al [24], which assessed the impact of OHI on patient-physician partnerships. Respondents also evaluated the overall usefulness of ChatGPT health information on a scale from poor (1) to excellent (5), and compared the usefulness of Chat-GPT derived OHI to information from other OHI sources and from their physicians [23].

Perceived barriers included the ability to obtain information in one’s preferred language, concerns about the accuracy of ChatGPT OHI, and methods of information verification. Previous use of ChatGPT for nonmedical purposes, the intended beneficiary of OHI (self vs close contact), and the type of concerns were assessed as “Cues to Action” [34].

Finally, we assessed behavioral outcomes which included the frequency of use, the timing of initial use serving as an indicator of early adoption, and the motivations behind utilization. These motivations included information-seeking for self-management, as a prelude to escalating care to a health professional, identifying alternative treatment, obtaining alternative information following a recent consultation, or simply pursuing interest or curiosity. Respondents were also asked if, as a result of their most recent use of ChatGPT, they asked a doctor for clarification, refused or requested a test or referral, changed medications; scheduled or canceled a doctor’s appointment, or performed no action. A final question was asked about sharing ChatGPT OHI with their physician.

All 48 questions (if applicable) were marked as required to answer to limit missing data, and respondents were unable to return to prior sections once they advanced.

### Pilot-Testing

After 3 rounds of internal revisions, we piloted within the researchers’ networks to enhance clarity, readability, and conciseness. This preliminary testing involved 15 community members connected to the researchers, including physicians, mental health therapists, and medical students with diverse demographic characteristics. Reviewers provided written and verbal feedback regarding ambiguity and challenges during the completion of the survey. Particular attention was paid to the relevance, appropriateness, and cognitive load required to answer items. We incorporated changes to the survey during a structured debriefing session. Based on this feedback, we excluded the other 4 eHealth inquiries, including “select all that apply” phrasing for questions regarding behavioral changes and reasons for initiation of ChatGPT use and incorporating priming subsection headings to prepare respondents for each varied task.

### Survey Population

The open survey was distributed to 21,499 members of ResearchMatch—a disease-neutral, Web-based recruitment registry—to help contact patients who have registered for eligibility to participate in clinical research studies [35]. Its 152,000 community reside in the Continental US and Puerto Rico, including those of all ages and races, both those in good health and those with health issues. ResearchMatch is supported by the US National Institutes of Health as part of the Clinical Translational Science Award program and is operated by the Vanderbilt University Medical Center, which maintains a large population of volunteers who have consented to be contacted by researchers about health studies for which they may be eligible. Participating health care systems around the country provide voluntary invitations for community members to join and make an account that allows for advertisements of available research studies. This population may preselect for a population with more dependable access to the internet such as White individuals with higher education and income. Platform moderators report that the majority of volunteers are female (69.1%, n=105,032), older than 18 years (97.2%, n=147,744), and White (70.6%, n=107,312) with 40.5% (n=61,560) volunteers reporting having no medical conditions. The platform allows researchers to specify cohorts prior to distribution by age, gender, race, health issue, and location. However, for the purpose of this study, the only parameters used were age to ensure adult participation only (≥18 years) and selection of all 50 US States with the exclusion of Puerto Rico. To reach the 21,499 members, researchers invited respondents in 1000 and 1499 (maximum) respondent batches for a total of 11 outreach rounds over the course of 2 months.

### Survey Administration

From June 10, 2023, to August 10, 2023, the survey was administered to consenting ResearchMatch members aged 18 years and older, with initial messaging being the only contact with participants. Initial contact with participants occurred within the internet-based contact platform within Research Match, wherein they were given a short message explaining that medical researchers investigating OHI were inviting them to participate in a research study. A link was attached to this email that allowed them to access the informed consent sheet and, upon consent, the survey. ReCAPTCHA technology was used to prevent bot survey abuse; however, no IP or cookie tracking was utilized to prevent duplicate entries. Informed consent for the survey was obtained from all participants as a cover page displayed prior to consent attestation. No personal identifying data was collected. The researchers incentivized participants to participate by offering them the opportunity to win one of 2 US $50 gift cards. A separate link to a Qualtrics form was used, and 2 respondents were randomly selected for each prize. Of the 21,499 participants given access to the survey link, 2406 participants completed the survey resulting in a response rate of 11.2%.

### Data Analysis

Statistical analysis was performed with Stata Statistical Software (release 18; StataCorp LLC). Descriptive statistics were used to characterize the cohort. In addition, significance testing using *t* tests and Pearson chi-square tests was used to evaluate the differences between users of ChatGPT OHI and nonusers. Results were considered statistically significant at *P*<.05. All *P* values were 2-sided. No methods to weight items or propensity scores were used to adjust the nonrepresentative sample. Presumably, due to the raffle incentive, there was little missing data. Missing observations were simply excluded from individual analyses without imputation.

## Results

### How Do ChatGPT Users Compare to Nonusers?

Table 1 depicts the demographic and health characteristics of all respondents, ChatGPT users, and ChatGPT nonusers. Among all respondents, most were female, White, and at least college-educated, and had less than US $100,000 in annual household earnings. The average age was 37.6 years. Most respondents had a continuity-based usual source of care such as a doctor’s office, health center, or VA, but a substantial number used urgent care centers or emergency departments. Consistent with the ResearchMatch sampling frame (individuals interested in participating in medical research), almost two-thirds rated their health as less than very good.

Among respondents, 517 (21.5%) were ChatGPT users and 1889 (78.6%) were nonusers. ChatGPT users were significantly younger than nonusers (32.8 vs 39.1 years, *P*<.001; Table 2). Compared with nonusers, ChatGPT OHI users were significantly more likely to identify as White (83.4% vs 78.6%, *P*=.02), earn less than US $100,000 annually (84.7% vs 73.1%, *P*<.001), and report educational attainment of less than a bachelor degree (50.1% vs 33%, *P*<.001, Table 1). Users were also more likely to use convenience or emergency (noncontinuity based) health care (52.2% vs 36.4%, *P*<.001), grade their health as less than very good (71.9% vs 63.8%, *P*=.001), and report heavy general OHI seeking frequency of weekly or more in the last 6 months (60.5% vs 49.3%, *P*<.001).

The Cronbach α for the scale measuring eLiteracy scores was 0.773, indicating good internal consistency among the items. Compared with nonusers, ChatGPT users displayed considerably lower eLiteracy scores (*P*=.007).

**Table 1:** Cohort demographic and health characteristics stratified by use of ChatGPT online health information (OHI).

Characteristic		All respondents (n=2406)	ChatGPT users (n=517)	Nonusers (n=1889)	*P* value
Age (years), mean (SD)		37.64 (13.76)	32.76 (7.00)	39.13 (14.92)	.001
**Sex (second cycle, n=137)**					
	Male	28 (20.4)	—^a^	—	—
	Female	101 (73.7)	—	—	—
	Other	8 (5.8)	—	—	—
**Race**					.02
	White	1834 (79.7)	431 (83.4)	1403 (78.6)	
	Non-White	468 (20.3)	86 (16.6)	382 (21.4)	
**Preferred language**					.21
	English	2244 (97.5)	500 (96.7)	1744 (97.7)	
	Non-English	58 (2.5)	17 (3.3)	41 (2.3)	
**Annual household income (US $)**					<.001
	Less than 100,000	1742 (75.7)	438 (84.7)	1304 (73.1)	
	100,000 or more	560 (24.3)	79 (15.3)	481 (27.9)	
**Education level**					<.001
	Less than BA/BS	848 (36.8)	259 (50.1)	589 (33)	
	BA/BS or greater	1454 (63.2)	259 (49.9)	1196 (67)	
**Preferred health care location**					<.001
	Continuity-based care (doctor’s office, health center, or VA^b^)	1345 (58.4)	247 (47.8)	1098 (58.1)	
	Transient care (emergency department, urgent care, store clinic, or multiple)	957 (41.6)	270 (52.2)	687 (36.4)	
**Health rating**					.001
	Good to poor	1510 (65.6)	372 (71.9)	1138 (63.8)	
	Very good to excellent	791 (34.4)	145 (28.1)	646 (36.2)	
**6-month OHI seeking frequency**					<.001
	Weekly or more	1183 (51.8)	313 (60.5)	870 (49.3)	
	Monthly or less	1099 (48.2)	204 (39.5)	895 (50.7)	
Average eLiteracy Score (Cronbach α=0.773^c^), mean (SD)		3.82 (0.01)	3.75 (0.03)	3.84 (0.02)	.007

^a^Not available.

^b^VA: Veteran's Affairs.

^c^Generated score is an average based on scores from 4 selected eLiteracy items measured from strongly disagree (1) to strongly agree (5)

**Table 2:** Usage characteristics of ChatGPT online health information (OHI) users.

Characteristic (n=517)		Values, n (%)
Prior use of ChatGPT		439 (86.4)
**Initiator of ChatGPT OHI use^a^**		
	Health care provider recommendation	224 (43.3)
	Advertisement on website or app	216 (41.8)
	Sponsored post or advertisement on social media	192 (37.1)
	Search engine result	181 (35)
	Friend or family recommendation for OHI use	179 (34.6)
	Health website or web-based forum	120 (23.2)
	News article or publication	109 (21.1)
	Expansion of previous non–health-related ChatGPT use	100 (19.3)
**Reasons for using ChatGPT for health information^a^**		
	Seeing if going to a health professional was necessary	245 (47.4)
	Looking for additional or alternative treatment options	239 (46.2)
	Clarifying or checking information given by a health professional	202 (39.1)
	Limited time or insufficient information during a health consultation	194 (37.5)
	Seeing if self-management is possible	181 (31)
	Disagreed with health professional and wanted a different information source	97 (18.8)
	Just out of interest	66 (12.8)
	Other	12 (2.3)
**Use frequency of ChatGPT OHI**		
	Once a month or less	68 (13.2)
	More than monthly but less than once a month	91 (17.6)
	About once a week	140 (27.1)
	2-3 times a week	153 (29.6)
	4-6 times a week	40 (7.7)
	Daily or almost daily	10 (1.9)
	Not applicable or single-use	6 (1.2)

^a^Respondents were allowed to select more than one response.

### How Do ChatGPT OHI Users Characterize the Purpose and Frequency of Their Use?

Most ChatGPT users endorsed a prior nonmedical use of the technology, with use initiation being primarily influenced by health care provider (HCP) recommendations, advertisements on websites or apps, and sponsored posts or ads on social media (Table 2). The most cited reasons for initiating use were determining the necessity of visiting a health professional and exploring alternative treatment options.

About a quarter reported using ChatGPT for OHI for 6 months or longer, which aligns with the timeline of ChatGPT’s introduction to the public. Use of ChatGPT OHI was frequent, with 40% (n=206) of respondents reporting use 2-3 times weekly or more.

### How Do Users Characterize the Ease, Understanding, and Usefulness of ChatGPT OHI?

Almost all users could obtain health information from the tool in their preferred language (Table 3). Users were divided regarding the overall usefulness of ChatGPT OHI; however, 63% (n=317) considered this information to be better than other OHI sources. Moreover, 4 of 5 users deemed ChatGPT OHI to be at least as good an information source as their physician.

The Cronbach α for the user experience scale was 0.67, indicating acceptable internal consistency. Respondents reported a generally positive ChatGPT experience (mean of 3.74 on 5-point scale). Conversely, 68% of users suspected that some aspect of the received information from the AI was inaccurate.

**Table 3:** User experience characterization with ChatGPT online health information (OHI).

Characteristic (n=502)		Values
Able to obtain OHI from ChatGPT in preferred language, n (%)		493 (97.6)
User experience scale (ease, understanding, relevance), mean (SD)^a^		3.74 (0.03)
**Overall usefulness of ChatGPT OHI, n (%)**		
	Poor to good	260 (51.8)
	Very good to excellent	242 (48.2)
**Usefulness compared with other OHI sources, n (%)**		
	Worst to much worse	48 (9.6)
	Same as other OHI sources	112 (24.3)
	Better to much better	317 (63.2)
**Usefulness compared with information from MD^b^ (better/much better), n (%)**		
	Worst to much worse	71 (14.1)
	Same as MD	209 (41.6)
	Better to much better	198 (39.4)
Suspected inaccuracy, n (%)		336 (67.9)
Presented information to doctor, n (%)		349 (67.5)

^a^Generated score is an average of scores from 3 questions pertaining to ease of use, understanding, and relevance of ChatGPT OHI, measured from Strongly Disagree (1) to Strongly Agree (5); (Cronbach α=0.645).

^b^MD: medical doctor.

### What Are the Most Common Health Behaviors That Follow the Use of ChatGPT?

Users commonly presented results of ChatGPT OHI to a physician (68%, n=338). Moreover, as a result of ChatGPT OHI use, 42.9% (n=222) of users asked a doctor for clarification of information, 45.8% (n=237) for more information, 35.6% (n=184) for a specialty referral, and 31% (n=160) for a new or different prescription (Table 4).

**Table 4:** Behavioral implications of ChatGPT online health information (OHI) use stratified by use frequency (respondents were allowed to select more than one response).

Characteristic (n=502)		Values, n (%)
**Behavior changes based on ChatGPT OHI**		
	Asked MD^a^ for more information	237 (45.8)
	Asked MD for clarification	222 (42.9)
	Requested a test or referral	184 (35.6)
	Self-medication/changed medication	160 (31)
	Refused tests/meds	103 (19.9)
	Scheduled appt	120 (23.2)
	Canceled appt	79 (15.3)
	No action taken	54 (10.4)

^a^MD: medical doctor.

## Discussion

### Principal Findings

This study is among the first to appraise patient use of ChatGPT-derived OHI. In this sample of participants in a national research cooperative, the use of ChatGPT for medical purposes was common, with users of the tool more likely to be White, have lower educational attainment, be in poor health, and receive care from noncontinuity based sources such as urgent care centers, retail clinics, and emergency departments. A large portion of users initiated use at the suggestion of a HCP and use of the tool was associated with altering appointments, changing medications, and consulting a physician as a result of their search.

Given the platform’s recent introduction, adoption by over one-fifth of the sample is remarkable. While the highly engaged ResearchMatch population may be inherently more likely to use new forms of OHI, ChatGPT appears poised for adoption by a growing proportion of the 81% of American adults who use OHI [36]. Moreover, the low response rate of this sample may limit the generalizability of the use patterns we observed.

The demographic profile of ChatGPT users reveals a possible digital divide in the realm of AI-driven OHI. Consistent with past studies depicting greater use of health information technologies by Whites compared with non-Whites [37,38], our findings suggest an inequity in the adoption of this source of OHI by minorities which may be explained by underserved minority individuals being more likely to have lower health literacy and less access to the internet [39-42].

Although heavier use of ChatGPT among respondents with lower educational attainment may challenge the standard view linking educational achievement, high digital literacy, and the use of health technology, it could be that ChatGPT’s conversational format is more accessible to individuals without college or graduate degrees than other OHI sources [43-45]. Conversely, it is possible that the relative lack of use amongst the more educated is due to a possible distrust of the platform or, more conservatively, a tapered approach to adoption advised by a greater ability to assess the efficacy of the tool for OHI. Still, it is also possible that this finding is idiosyncratic, a function of our nonrepresentative survey sample.

ChatGPT OHI users were also found to have lower eHealth literacy, a finding that is seemingly paradoxical. However, one plausible interpretation of the finding that ChatGPT users have both lower eHealth literacy and lower educational attainment than their nonusing counterparts is less sophisticated health care consumers may benefit differentially from the conversational format of ChatGPT, while more educated and internet-savvy individuals may be able to satisfy their information needs in other ways (such as the use of traditional search engines, subscriptions to health education blogs such as the Harvard Health Letter, etc).

The preference of individuals in poorer health for utilizing ChatGPT as a health information resource aligns with the broader understanding that individuals with more significant health challenges may have increased information needs [46,47]. This heightened demand may stem from the necessity to understand and manage complex health conditions, leading to a more active pursuit of diverse information sources, including AI platforms like ChatGPT. Moreover, as ChatGPT users prefer transient health care more often, those accustomed to expedient, on-demand care are likely more inclined to gravitate towards ChatGPT OHI’s mode of instantaneous personalized information [46]. Moreover, as increased OHI use is associated with barriers to traditional health care access, it is likely that when faced with barriers to health care—such as cost, availability, and accessibility—individuals turn to alternative information sources, including ChatGPT [48]. This presents physicians with a unique opportunity to identify and counsel patients about the value and limitations of using LLMs for health information, offering to be available in the appropriate context (eg, a future office visit) to help interpret the information received. This partnership not only bridges information gaps but also reinforces the physician’s role in collaborating with patients as they navigate their health information journey. Moreover, as physicians become more familiar with the abilities and inabilities of ChatGPT and similar programs, they can more effectively counsel patients on the prudent use of such AI resources in complementing ongoing medical care.

While most users came to the tool through advertisements or social media posts, a substantial number reported using it at the suggestion of HCPs. The percentage of physicians who see digital health tools as an advantage for patient care grew from 85% in 2016 to 93% in 2022, corresponding to a similar increase use of digital health tools by physicians [49]. Moreover, as approximately 10% of US HCPs endorse the use of AI, with another 50% considering future use, HCPs’ personal experiences with ChatGPT may influence their willingness to recommend them to patients, reflecting a growing confidence in the utility of such AI tools for patient care [50]. Considering that almost two-thirds of ChatGPT users reported ChatGPT use once a week or more, many respondents likely trusted their HCP’s recommendation. However, it is important to clarify that this survey item instructed respondents to pick this option as a reason for the recommendation, even if the HCP was a family member or friend. This may have augmented the number of individuals who report having been recommended the tool by a care provider. A poor understanding of who may qualify as a HCP may also account for this unexpectedly high referral rate, considering the novelty and skepticism surrounding ChatGPT OHI. Nonetheless, patient awareness and confidence in ChatGPT within this study have arguably outpaced the completion of rigorous studies of the tool’s efficacy and accuracy in delivering health recommendations.

For about half of respondents, ChatGPT use was followed by some health behavior such as formalized care-seeking, asking for information, and asking for further action, including setting or canceling an appointment or requesting new testing. OHI has been shown in multiple studies to stimulate care-seeking and clinical question-asking [51,52]. It is remarkable, however, that ChatGPT, despite its relative infancy, appears to promote behavioral change rates similar to more established sources of OHI. Further, given that most patients reported care-seeking behaviors despite also believing some aspect of the information received was inaccurate, it is promising that verifying the information with a physician arose as a leading resultant health behavior. This aligns with previous studies that emphasize the role of physicians in verifying traditional OHI, suggesting that the traditional physician-patient relationship may endure as physicians who can identify likely ChatGPT OHI users will be able to counsel regarding the efficacy and accuracy of the information received [53,54].

User perceptions of their ChatGPT OHI experience along the dimensions of comparable usefulness, ease of use, and suspected inaccuracy raise several intriguing contrasts. Around 52% (n=260) of respondents rated ChatGPT to be overall poor to good in usefulness, while more than 80% (n=429) rated it to be better than or as good as other OHI sources. Users may appreciate certain aspects of ChatGPT, such as the conversational interaction, user interface, and quick reception of information, which may not be as easily executed on other OHI platforms. It may be the case that users who rated in this manner more heavily weighted the advantages of ChatGPT’s information delivery and accessibility over their perception of inaccuracy or information usefulness. Further, the dichotomy between the concerns for inaccuracy and the somewhat favorable ratings on usefulness suggests a nuanced understanding of “usefulness” by the users. While our data may suggest action is likely after the use of ChatGPT, many patients may find value in ChatGPT as a starting point for health information or as a means to facilitate discussions with HCPs, a relationship past research has supported for traditional OHI [53,54].

Thus, during the digital health information age, a balance must be held between the desire for rapid information acquisition and the need for accurate, trustworthy advice. Even more importantly, it is important to consider user information-seeking experience when evaluating these OHI tools, as this may play an important role in the uptake and appraisal of the tools’ usefulness to patients.

### Limitations and Future Directions

While shedding light on the adoption and utilization of ChatGPT for OHI, this study carries 4 principal limitations. First, the study’s cross-sectional design limits the understanding of causal relationships and does not capture behavior over time. Rather than prompting particular health-related decisions or behaviors, the use of ChatGPT may instead have been a consequence of preexisting decisions or behaviors. Second, as a self-administered survey, this study is subject to reporting and recall bias. Third, two features limit the generalizability of the results: (1) the ResearchMatch population skews younger, less ethnically diverse, and arguably more engaged with health and health care than the US population as a whole; and (2) despite similar demographic profiles, our sample may differ from the ResearchMatch population in other (unmeasured) ways [55]. These differences may have influenced perceptions and experiences with ChatGPT in the OHI context. Future investigations should focus on diverse sampling strategies to include participants of varying degrees of education and digital literacy to examine how different populations engage with ChatGPT for OHI. Fourth, we could not directly examine the specific content accessed by ChatGPT users nor the completeness or accuracy of the information received.

### Conclusions

This study revealed a swift adoption of ChatGPT, particularly among younger patients with poorer health and those using transient (noncontinuity-based) forms of health care. As ChatGPT appears to influence both intensity and types of care-seeking behavior, physicians and other HCPs must proactively identify and counsel patients on best practices for the use of this emerging technology. This skill will be vital in preserving the integrity of the physician-patient relationship and ensuring safe and effective health care in an increasingly AI-driven digital world. More research is needed to understand how patients and physicians can work together to make optimal use of these powerful but potentially hazardous tools.

## Appendix

Multimedia Appendix 1 REDCap (Research Electronic Data Capture) questionnaire.

Multimedia Appendix 2 Health behaviors model for uptake and adherence to ChatGPT health information.

## Abbreviations

AI: artificial intelligence
HCP: health care provider
LLM: large language model
OHI: online health information
REDCap: Research Electronic Data Capture
