# Supplementary material for: Characterizing the Adoption and Experiences of Users of Artificial Intelligence–Generated Health Information in the United States: Cross-Sectional Questionnaire Study
Source: J Med Internet Res. 2024 Aug 14;26:e55138. doi: 10.2196/55138 (PMC11358651; doi:10.2196/55138)
Supplement: Multimedia Appendix 1 [file jmir_v26i1e55138_app1.pdf]

Data Dictionary Codebook

Early Adopter's Use of AI Generated Information (General 2 with ResearchMatch) (PID: 11481)

03/12/2023 9:03pm

#	Variable / Field Name	Field Label<br>*Field Note*	Field Attributes (Field Type, Validation, Choices, Calculations, etc.)
**Instrument: ChatGPT Consent Page (chatgpt_consent_page)** Enabled as survey			
1	[record_id]	Record ID	text
2	[consent_1]		descriptive
3	[consent_3]	By completing the survey, you are agreeing to participate in the research. Please select "Agree" if you are agreeing to take part in this study.	radio<br>1 Agree<br>2 Disagree<br><br>Stop actions on 2
4	[ty]	Thank you to agreeing to participate. Please click "Submit" to proceed to the survey.	descriptive
5	[chatgpt_consent_page_complete]	Section Header: *Form Status*<br>Complete?	dropdown<br>0 Incomplete<br>1 Unverified<br>2 Complete
**Instrument: Chatgpt Questions Nonmh (chatgpt_questions_nonmh)** Enabled as survey			
6	[gender]	Section Header: *First, for statistical purposes, we want to learn a few things about you. Your information will be combined with other survey participants so that we know something about the people taking part in this study.*<br>What is your gender?	radio<br>1 Female<br>2 Male<br>3 Transgender man<br>4 Transgender woman<br>5 Gender-fluid/Non-conforming<br>6 Don't know<br>7 Prefer not to answer.
7	[age]	What is your age	text
8	[race]	What is your race?	radio, Required<br>1 White<br>2 Black or African American<br>3 American Indian or Alaska Native<br>4 Hispanic or Latino<br>5 Asian<br>6 Native Hawaiian or Other Pacific Islander<br>7 Some other race (do not identify with any one of the race categories

			above)
9	[language]	What is your preferred language? (reading and writing)	radio, Required<br>1 English<br>2 Spanish<br>3 Other (please specify)
10	[language_other]<br>Show the field ONLY if:<br>[language] = '3'	Language	text, Required
11	[income]	What is your estimated total annual household income?	radio, Required<br>1 Less than $25,000<br>2 $25,000 to $34,999<br>3 $35,000 to $49,999<br>4 $50,000 to $74,999<br>5 $75,000 to $99,999<br>6 $100,000 to $149,999<br>7 $150,000 or more
12	[education]	What is the highest level of education that you have completed?	radio, Required<br>1 Some high school<br>2 High school graduate<br>3 Some college<br>4 Associate degree<br>5 Bachelor's degree<br>6 Master's degree<br>7 Ph.D., law or medical degree<br>8 Other advanced degree beyond a Master's degree
13	[care_location]	Do you have a place that you USUALLY/MOST OFTEN go to if you are sick and need health care?	radio, Required<br>1 A doctor's office or health center<br>2 Urgent care center or clinic in a drug store or grocery store<br>3 Hospital emergency room<br>4 A VA Medical Center or VA outpatient clinic<br>5 Some other place<br>6 I don't go to any one place most often<br>7 Don't Know
14	[health]	How would you rate your health? Please indicate whether you consider your health to be	radio, Required

		excellent, very good, good, fair, or poor.	1 Poor<br>2 Fair<br>3 Good<br>4 Very good<br>5 Excellent
15	[eliteracy_q4]	Section Header: *For the next 5 questions, please rate your level of agreement from "strongly disagree" to "strongly agree".Health resources are sources that provide a way to get information about illnesses, find support and connect with others such as online support communities, social media, websites from peers/health professionals etc.)*<br>I know where to find helpful health resources on the Internet	radio<br>1 strongly disagree<br>2 disagree<br>3 agree nor disagree<br>4 agree<br>5 strongly agree
16	[eliteracy_q5]	I know how to use the health information I find on the Internet to help me	radio<br>1 strongly disagree<br>2 disagree<br>3 agree nor disagree<br>4 agree<br>5 strongly agree
17	[eliteracy_q7]	I can tell high quality from low quality health resources on the Internet	radio<br>1 strongly disagree<br>2 disagree<br>3 agree nor disagree<br>4 agree<br>5 strongly agree
18	[eliteracy_q8]	I feel confident in using information from the Internet to make health decisions	radio<br>1 strongly disagree<br>2 disagree<br>3 agree nor disagree<br>4 agree<br>5 strongly agree
19	[ohi_seeking]	Section Header: *The purpose of the next set of questions is to learn about your use of online health information sources.*<br>On average, how often did you seek online health information in the last 6 months?	radio, Required<br>1 Daily<br>2 Several times a week<br>3 Weekly<br>4 Monthly<br>5 Rarely<br>6 Never<br><br>Stop actions on 6

20	[ohi_tools]	Section Header:<br>Which of the following kinds of tools have you used to seek online health information? (select all that apply)	checkbox, Required<br>1 ohi_tools___1 Internet search engines (e.g.Google, Bing)<br>2 ohi_tools___2 Online Encyclopedia sites (e.g. Wikipedia)<br>3 ohi_tools___3 Online professional health sites (e.g., WebMD, MedlinePlus, MayoClinic)<br>4 ohi_tools___4 ChatGPT<br>5 ohi_tools___5 Online forums (e.g. Reddit subgroups, Facebook groups, specialized organization forums such as American Heart Association patient forum)<br>6 ohi_tools___6 Q&A sites (Quora, Yahoo Answers, etc)<br>7 ohi_tools___7 Other (please specify)
21	[language_other_2]<br>Show the field ONLY if:<br>[ohi_tools(7)] = '1'	Other tools used:	text
22	[attention_1]<br>Show the field ONLY if:<br>[ohi_tools(4)] = '1'	Section Header: *The next series of questions will explore your use of ChatGPT.*<br>Please select "strongly agree" as the answer for this survey item to demonstrate your attention.	radio, Required<br>1 Agree<br>2 Disagree<br>3 Strongly agree<br>4 Strongly disagree
23	[general_gptuse]<br>Show the field ONLY if:<br>[ohi_tools(4)] = '1'	Before using ChatGPT to obtain online health information, had you used ChatGPT for any other purpose?	radio, Required<br>1 Yes<br>2 No
24	[gptuse_prompt]<br>Show the field ONLY if:<br>[ohi_tools(4)] = '1'	Which of the following prompted you to use ChatGPT for online health information? Please select all that apply.	checkbox, Required<br>1 gptuse_prompt___1 A friend or family member who has used the tool for health information before recommended it to me

			<table><tr><td>2</td><td>gptuse_prompt___2</td><td>I saw an advertisement for the tool on a website or mobile app</td></tr><tr><td>3</td><td>gptuse_prompt___3</td><td>I saw a sponsored post or ad for the tool on social media</td></tr><tr><td>4</td><td>gptuse_prompt___4</td><td>A healthcare provider recommended the tool (for family/friend in the health profession, choose this option)</td></tr><tr><td>5</td><td>gptuse_prompt___5</td><td>I found the tool through a specific search engine (e.g. Google, Bing, Yahoo)</td></tr><tr><td>6</td><td>gptuse_prompt___6</td><td>I found the tool through a health-related website or online forum</td></tr><tr><td>7</td><td>gptuse_prompt___7</td><td>I learned about the tool from a news article or health-related publication</td></tr><tr><td>8</td><td>gptuse_prompt___8</td><td>I had previous experience with the tool and expanded my use to health information.</td></tr><tr><td>9</td><td>gptuse_prompt___9</td><td>Other (please specify)</td></tr></table>
25	[gptuse_fr]<br><br>Show the field ONLY if:<br>[gptuse_prompt(9)] = '1'		notes

26	[duration]<br><br>Show the field ONLY if:<br>[ohi_tools(4)] = '1'	How long have you been using ChatGPT for the purpose of seeking online health information (excluding all other uses)?	radio, Required<br>1 Less than a month<br>2 1-3 months<br>3 3-6 months<br>4 6 months or longer<br>5 N/A; have used it just once.
27	[frequency]<br><br>Show the field ONLY if:<br>[ohi_tools(4)] = '1'	How often do you use ChatGPT specifically for seeking online health information (excluding all other uses)?	radio, Required<br>1 Once a month or less<br>2 More than monthly but less than once a week<br>3 About once a week<br>4 2-3 times a week<br>5 4-6 times a week<br>6 Daily or almost daily<br>7 N/A; have used it just once.
28	[behalf]<br><br>Show the field ONLY if:<br>[ohi_tools(4)] = '1'	Section Header: *The next set of questions refer to your use of ChatGPT for online health information in general.*<br><br>Most often, you use ChatGPT to find online health information for...	radio, Required<br>1 Myself<br>2 Someone close to me<br>3 Myself and someone close to me<br>4 Something not specific to me or someone close to me
29	[issue]<br><br>Show the field ONLY if:<br>[ohi_tools(4)] = '1'	Generally, for what type of issue do you seek online health information on ChatGPT?	radio, Required<br>1 A new health issue<br>2 A long-standing health issue<br>3 Both a new and long-standing health issue<br>4 A general public health issue<br>5 Other (please specify)
30	[issue_fr]<br><br>Show the field ONLY if:<br>[issue] = '5'		notes
31	[gptuse_reason]<br><br>Show the field ONLY if:<br>[ohi_tools(4)] = '1'	In your experience, what have been some reasons for turning to ChatGPT as a source of online health information? Please select all that apply.	checkbox, Required<br>1 gptuse_reason___1 I wanted to see if I could manage the issue by myself.<br>2 gptuse_reason___2 I wanted to see if going to a health professional was necessary.

			3 gptuse_reason___3 I wanted to look fo additional/alternati treatment options.<br>4 gptuse_reason___4 I wanted to clarify o check information that had been give by a health professional.<br>5 gptuse_reason___5 There was limited time or insufficient information during meeting with a health professional for the additional questions I had.<br>6 gptuse_reason___6 I disagreed with certain points mad by a health professional and wanted a different information source<br>7 gptuse_reason___7 Just out of interest.<br>8 gptuse_reason___8 Other (please specify)
32	[gptuse_reasonfr]<br>Show the field ONLY if:<br>[gptuse_reason(8)] = '1'		notes
33	[severity]<br>Show the field ONLY if:<br>[ohi_tools(4)] = '1'	Typically, how serious do you think a health concern is before you turn to ChatGPT for online health information?	radio, Required<br>1 Not at all serious<br>2 Slightly serious<br>3 Moderately serious<br>4 Very serious<br>5 Extremely serious<br>6 Not sure/not applicable to most recent use
34	[presentedlang]<br>Show the field ONLY if:<br>[ohi_tools(4)] = '1'	Are you able to obtain the online health information from ChatGPT in your preferred language?	radio, Required<br>1 Yes<br>2 No
35	[ease]<br>Show the field ONLY if:<br>[ohi_tools(4)] = '1'	Section Header: *For the next set of questions, please state your level of agreement with each statement for your use of ChatGPT for online health information.*<br>It is easy to use ChatGPT for the purpose of getting online health information. (eg. Log in, navigation etc)	radio, Required<br>1 strongly disagree<br>2 disagree<br>3 agree nor disagree

			4 agree<br>5 strongly agree
36	[relevance]<br>Show the field ONLY if:<br>[ohi_tools(4)] = '1'	The health information I receive from ChatGPT is relevant to my specific needs.	radio, Required<br>1 strongly disagree<br>2 disagree<br>3 agree nor disagree<br>4 agree<br>5 strongly agree
37	[understand]<br>Show the field ONLY if:<br>[ohi_tools(4)] = '1'	The health information received from Chat GPT is easy to understand.	radio, Required<br>1 strongly disagree<br>2 disagree<br>3 agree nor disagree<br>4 agree<br>5 strongly agree
38	[useful]<br>Show the field ONLY if:<br>[ohi_tools(4)] = '1'	How would you rate the usefulness of the health information you received on ChatGPT?	radio, Required<br>1 Poor<br>2 Fair<br>3 Good<br>4 Very good<br>5 Excellent
39	[usefulohi]<br>Show the field ONLY if:<br>[ohi_tools(4)] = '1'	How does the usefulness of the health information you found on ChatGPT compare to the information you receive from other sources of online health information?	radio, Required<br>1 Much worse than other sources<br>2 Worse than other sources<br>3 Same as other sources<br>4 Better than other sources<br>5 Much better than other sources<br>6 Not sure/No opinion
40	[usefulmd]<br>Show the field ONLY if:<br>[ohi_tools(4)] = '1'	How does the usefulness of the health information you found on ChatGPT compare to the information you receive from your doctor?	radio, Required<br>1 Much worse than the information from your doctor<br>2 Worse than the information from your doctor<br>3 Same as the information from your doctor<br>4 Better than the information from your doctor<br>5 Much better than the information from your doctor<br>6 Not sure/No opinion

41	[inacc]<br><br>Show the field ONLY if:<br>[ohi_tools(4)] = '1'	Section Header: *This is the last page of survey questions. Thanks for your participation.*<br><br>Have you ever suspected that the information provided by ChatGPT was inaccurate?	radio<br>1 Yes<br>2 No
42	[verification]<br><br>Show the field ONLY if:<br>[ohi_tools(4)] = '1'	When you get online health information from ChatGPT, which of the following methods do/did you generally use to verify the quality of the information? (Select all that apply)	checkbox, Required<br>1 verification___1 Ask my doctor<br>2 verification___2 Ask other health care professionals<br>3 verification___3 Ask friends and family<br>4 verification___4 Use other online sources of information (please specify)<br>5 verification___5 Read books or articles written by physicians or healthcare professionals<br>6 verification___6 Other (please specify)<br>7 verification___7 None, I do not generally verify the accuracy of the information
43	[altsource]<br><br>Show the field ONLY if:<br>[verification(4)] = '1'	Other online sources of information used to verify:	notes
44	[altmeth]<br><br>Show the field ONLY if:<br>[verification(6)] = '1'	Other methods of verifying quality of information:	notes
45	[behavior]<br><br>Show the field ONLY if:<br>[ohi_tools(4)] = '1'	Based on the online health information you've gotten from ChatGPT, have you ever done the following? (select all that apply)	checkbox, Required<br>1 behavior___1 Asked a doctor for more information<br>2 behavior___2 Asked a doctor for clarification of information<br>3 behavior___3 Requested a test or referral from doctor<br>4 behavior___4 Self-medicated/Changed meds<br>5 behavior___5 Refused tests/meds

			6 behavior___6 Scheduled a doctor appointment<br>7 behavior___7 Canceled a doctor appointment<br>8 behavior___8 No action taken based on information from the most recent use<br>9 behavior___9 Other (please specify)
46	[behaviorfr]<br>Show the field ONLY if:<br>[behavior(9)] = '1'		notes
47	[sharemd]<br>Show the field ONLY if:<br>[ohi_tools(4)] = '1'	Have you ever shared health information from ChatGPT to your doctor during a visit?	checkbox, Required<br>1 sharemd___1 Yes<br>2 sharemd___2 No<br>3 sharemd___3 No but I wanted to<br>4 sharemd___4 No but I plan to in the future<br>5 sharemd___5 Not sure
48	[chatgpt_questions_nonmh_complete]	Section Header: *Form Status*<br>Complete?	dropdown<br>0 Incomplete<br>1 Unverified<br>2 Complete
