# Supplementary figures and images for: Characterizing the Adoption and Experiences of Users of Artificial Intelligence–Generated Health Information in the United States: Cross-Sectional Questionnaire Study
Source: J Med Internet Res. 2024 Aug 14;26:e55138. doi: 10.2196/55138 (PMC11358651; doi:10.2196/55138)

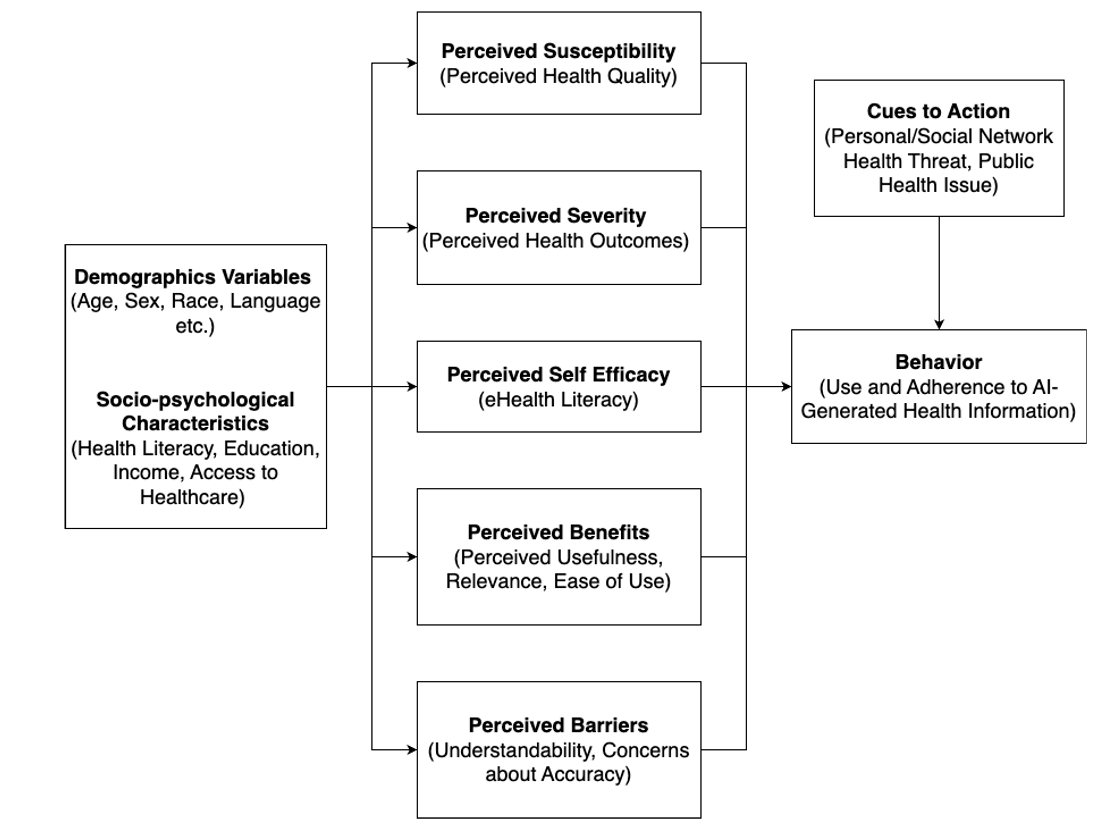

Supplement: Multimedia Appendix 2 [file jmir_v26i1e55138_app2.png]
